# Facilitators of and Barriers to Global Digital Oral Health: Mixed Methods Study

**DOI:** 10.2196/76236

**Published:** 2026-04-30

**Authors:** Elham Emami, Pascaline Kengne Talla, Camille Inquimbert, Nicolas Giraudeau

**Affiliations:** 1 Faculty of Dental Medicine and Oral Health Sciences McGill University Montreal, QC Canada; 2 Public Health Department, Faculty of Dental Medicine, Institut Desbrest d’épidémiologie et de santé publique, IDESP UMR UA11 INSERM University of Montpellier Montpellier, Occitanie France; 3 Centre d’études politiques et sociales, Centre national de la recherche scientifique University of Montpellier Montpellier France

**Keywords:** digital health, mobile health, mHealth, oral health, health policy, World Health Organization

## Abstract

**Background:**

Digital oral health builds on the broader framework of eHealth, leveraging digital technologies to improve patient care, increase access to dental services, and enhance oral health outcomes. However, health care organizations and institutions encounter challenges in implementing digital oral health interventions across various levels. Addressing these challenges requires a comprehensive understanding of the barriers and facilitators that influence its successful adoption.

**Objective:**

This study aimed to explore the facilitators of and barriers to the implementation of digital oral health programs from the perspective of chief dental officers from countries across the World Health Organization (WHO) regions.

**Methods:**

This study is part of a broader investigation into global readiness for digital oral health. Participants were the 144 chief dental officers or designated oral health officials within ministries of health across the 6 WHO regions. An explanatory sequential mixed methods design was used across 2 phases. In the quantitative phase, an online survey was administered using the WHO’s global survey on eHealth instrument. Some items were modified slightly to be applied to the field of dentistry. Descriptive statistics were used to present the quantitative data. In the qualitative phase, data were collected through virtual interviews, using an interview guide developed based on preliminary findings from the quantitative phase, the technology acceptance model, and the eHealth readiness assessment tool. The qualitative data were analyzed using thematic analysis.

**Results:**

The survey response rate was 70.1% (101/144). The qualitative phase involved in-depth interviews with 15 participants. The findings were integrated under 2 broad themes of facilitators and barriers. Perceived facilitators included the existence of national policies and guidelines on eHealth. Approximately 63.9% (53/83) of the respondents indicated the presence of a national oral health policy in their countries. Capacity building, motivation of health care providers and academic leadership, digital health training for students or professionals, and WHO support to implement the mOral Health program were the other facilitators. The strongest barriers were a lack of funding to develop and support digital health programs, lack of norms and standards to guarantee application interoperability, and lack of equipment and/or connectivity. Approximately 45.1% (37/82) of the participants reported having government-sponsored mobile health programs, while 31.7% (26/82) reported having no financial support for the implementation of national digital oral health programs. Furthermore, lack of evidence on the effectiveness and cost-effectiveness of programs was highlighted as a barrier by 73.8% (59/80) and 73% (57/78) of the participants, respectively.

**Conclusions:**

The results of this study enabled the identification of key barriers to and enablers of the implementation of digital oral health programs in WHO member countries. Supportive governmental policies and adequate funding and investment in digital infrastructure and technologies are essential to mitigate digital oral health–related challenges.

## Introduction

Digital health has been defined as “the cultural transformation of how disruptive technologies that provide digital and objective data accessible to both caregivers and patients lead to an equal level doctor-patient relationship with shared decision-making and the democratization of care” [1]. Digital health supports the Quintuple Aim [2] in health care by improving patients’ experiences and the population’s health, and increasing accessibility and equity in care while reducing the costs of the health care system [3]. This definition applies to dentistry as well, as oral health is an integral part of overall health [4]. In this context, digital oral health reflects the same principles, leveraging tools such as tele-dentistry, artificial intelligence (AI)–powered diagnostics, digital imaging, and electronic health records to enhance access, precision, and patient engagement in oral health care.

Over the past decade, the World Health Organization (WHO) has actively supported the integration of digital technologies into health care systems, most notably through its global strategy on digital health 2020-2025 [2]. Furthermore, the global strategy and action plan on oral health recognizes digital health interventions as catalysts to optimize oral health equity and increase environmental sustainability to achieve universal coverage. The WHO has also issued implementation guidelines for mobile technologies as part of the mOral Health Program [5]. Although digital innovations such as computer-aided design and manufacturing, digital imaging, and AI [5,6] have progressed rapidly in dentistry, their integration into oral health care systems has not kept the pace. This gap is influenced by several key factors. Among these, shared values, professional attitudes and behaviors, and organizational readiness, along with enablers such as technological infrastructure, workforce capacity, and available funds, are essential for the successful adoption and sustained use of digital oral health interventions [6,7].

The recent global COVID-19 pandemic has significantly accelerated the adoption of tele-dentistry across health care systems worldwide [7]. Faced with the urgent need to provide care while minimizing the risk of virus transmission, many dental practices and health care systems rapidly integrated telehealth technologies [7]. This shift allowed for the partial fulfillment of emergency dental care needs, enabling patients to receive timely consultations and guidance remotely [8,9]. Despite this progress, evidence indicates that the integration of digital technologies in oral health care organizations and institutions continues to face various barriers at the micro, meso, and macro levels and across the 4 domains of integration: clinical, professional, organizational, and systemic [10,11].

This indicates the need for the global scale-up of a digital oral health plan and a transformative shift in the delivery of dental care worldwide through the adoption of digital platforms that facilitate remote consultations, modernize electronic oral health care records and data management, and enable virtual and AI-driven diagnostic systems [12]. This global scale-up of the digital plan will only be successful if it is grounded in a system-based approach and if it is universally applicable to all countries. This also depends on international collaboration, universal standardization, and universal protocols [13]. This necessitates an understanding of organizational readiness, or “the degree to which users, healthcare institutions, and the healthcare system itself are prepared to participate and succeed with e-health implementation” [14-16]. The dimensions of e-readiness include governmental readiness, or the presence of relevant policies and funding; organizational readiness, or the presence of policies and management support; and, finally, societal readiness, or the interaction of health care institutions with their government and communities, as well as the readiness of health care providers and patients [17-19].

To understand the state of readiness of countries for digital oral health interventions and inform a comprehensive digital oral health strategy, a good understanding of global readiness, challenges, opportunities, capacities, and obstacles is required. Therefore, built on a strong partnership among the WHO, the University of Montpellier, and McGill University Faculty of Dental Medicine and Oral Health Sciences, and with the overarching goal of optimizing patient-centered care and value-based health care [20], a broad study on global readiness for digital oral health was conducted. This paper specifically addresses the following research question: *what are the barriers to and facilitators of implementing digital oral health programs as perceived by chief dental officers (CDOs) across WHO regions?*

## Methods

### Overview

This study is part of a broader study on global readiness for digital oral health, and it focuses on presenting the results regarding facilitators of and barriers to global digital oral health. The reporting of this study adheres to the GRAMMS (Good Reporting of A Mixed Methods Study) guidelines [21]. The GRAMMS checklist is available in Multimedia Appendix 1.

### Study Design and Methods

An explanatory sequential mixed methods [22] study was conducted in 2 phases. The initial phase used a quantitative approach. Subsequently, a qualitative phase was undertaken aimed at contextualizing and elaborating on the quantitative findings [23]. This design is valuable for gaining an in-depth understanding of the context of the study and supports policy implementation. The quantitative and qualitative data were collected sequentially from April 2022 to May 2023 but were analyzed simultaneously and in an iterative way to validate and confirm the results (triangulation) [24].

### Conceptual Frameworks

The technology acceptance model [25] and the eHealth readiness assessment tool by Khoja et al [17] were used as conceptual frameworks for the qualitative study. The technology acceptance model is a well-known theory widely used in research on health IT, explaining how factors such as perceived ease of use, perceived usefulness, and behavioral intention influence technology acceptance. The eHealth readiness assessment tool evaluates e-readiness among managers and health care providers across the categories of core, societal, and policy readiness, as well as technological readiness and learning readiness.

### Study Participants

The potential study participants were invited through a collaboration with the WHO. One of the study team members (NG), who acts as a WHO expert in digital health, facilitated the communication with CDOs via email. The invitation email included an informative letter summarizing the study objectives and methods, including the anonymous online survey. The email addresses we contacted were valid as none of the messages were returned as undeliverable.

The eligibility criteria were being a CDO or the responsible oral health officer in the ministry of health of countries that were WHO members and represented the 6 WHO regions. The 6 WHO regions encompass the African Region, the Region of the Americas, the Eastern Mediterranean Region, the European Region, the South-East Asia Region, and the Western Pacific Region. These countries varied with regard to income level and included high, upper-middle, lower-middle, and low income levels. The income level was defined using the World Development Indicators database gross national income per capita, which categorizes economies based on their gross national income per capita into different income groups [26]. The invitation was sent only to 144 CDOs or responsible officers from 6 WHO regions and 194 WHO member countries. The FDI World Dental Federation maintains a section dedicated to CDOs and dental public health worldwide.

### Data Collection

#### Phase 1: Quantitative Data

An online survey was conducted using the WHO’s global survey on eHealth instrument [27]. The items of this questionnaire were modified slightly to be applied to the field of dentistry without any change in the constructs, which would have required further validation. Translation services were used to translate the modified questionnaire into other languages according to the participants’ language preference.

The survey instrument comprised nine sections: (1) demographic characteristics of the participants, (2) digital health foundations, (3) mobile health (mHealth) and telehealth, (4) big data, (5) electronic health records, (6) use of e-learning in health sciences, (7) social media, (8) barriers to implementing digital health programs, and (9) digital health network. However, for this study, data related only to facilitators and barriers were included (Multimedia Appendix 2). These included responses on the existence of national digital and oral health policies, telehealth policies, mHealth programs, funding, infrastructure, and WHO support. In addition, items on training and the interoperability, effectiveness, and cost-effectiveness of the programs were included.

The modified version was pilot-tested among a group of clinicians and oral health administrators, who evaluated the relevance, length, and overall comprehensiveness of the items. To enhance the response rate, reminders were sent to participants 2 and 3 weeks after the initial distribution of the survey. Participants were also asked to indicate their willingness to take part in the qualitative phase of the study. Participants were asked to sign a consent form before filling out the survey, which was launched through the WHO platform. Participants who agreed to take part in the study were asked to indicate their preferred language for the survey.

#### Phase 2

One-on-one, virtual, face-to-face 60- to 90-minute interviews were conducted to collect the qualitative data. Maximum variation and purposeful sampling techniques were used to represent the WHO’s 6 regions and varying income levels [28]. This approach allowed for capturing the perspectives of participants from different countries with diverse policies, cultural contexts, socioeconomic backgrounds, and educational systems [28]. The interviews were conducted virtually using secure platforms such as Zoom (Zoom Video Communications), Webex Meetings (Cisco Systems), or Microsoft Teams, depending on the accessibility, preferences, and convenience of the study participants. The interviews were conducted in English or French as requested and selected by the study participants. They were carried out by 2 members of the research team (PKT and CI) fluent in both languages and with previous training and expertise in qualitative research. Using the study’s conceptual frameworks and the results of the quantitative study, an interview guide with open-ended questions was developed to collect “information-rich” data and capture perspectives on the facilitators and barriers related to the implementation of digital oral health solutions, including the mOral Health program [29] (Multimedia Appendix 3). Data collection and analysis were conducted concurrently. Interviews continued until saturation was reached to ensure that no new information would be obtained from further interviews [30]. All interviews were recorded and transcribed verbatim.

### Ethical Considerations

Ethics approval for this study was granted by the institutional review board (A04-E17-21A) of McGill University. Participation in the study was completely voluntary, and informed consent was obtained from all participants. Participants were clearly informed that any data collected during both the quantitative and qualitative phases would be deidentified to maintain confidentiality and ensure anonymity. No monetary or other forms of compensation were provided for participation.

### Data Analyses

#### Quantitative Phase

Non–English- or French-language surveys were translated into English. For the purpose of this study, only items relevant to the study objectives were analyzed. Surveys that did not specify the country of origin and those that were less than 90% complete were excluded. Furthermore, as there were minimal missing data, a modified complete case analysis was performed [31]. Descriptive statistics were used to present the data using the SPSS Statistics software (version 29.0.0.0; IBM Corp). Sample size estimation was not conducted for this descriptive study because the aim was to summarize and describe the facilitators and barriers rather than hypothesis testing or predictions about associations between variables.

#### Qualitative Phase

Thematic analysis was conducted using both deductive and inductive approaches, including data transcription, coding, categorization, analysis, and interpretation [30]. Data transcription was carried out through repeated examination of the recorded interviews by 2 researchers (PKT and CI). The research team engaged in regular, in-depth discussions to resolve uncertainties in coding and interpretation, reaching consensus to uphold the study’s credibility.

During the categorization phase, codes with similar conceptual content were grouped to identify emerging themes. Each stage was reviewed by 3 members of the research team (PKT, EE, and CI) and discussed. This approach facilitated shared interpretation, resolution of discrepancies, and consensus building around codes and themes. It also ensured trustworthiness and analytic accuracy, minimized individual bias and misinterpretation, and prevented the exclusion of any relevant data, thus strengthening the confirmability and credibility of the findings [32,33].

Data analysis was conducted manually, supported by the use of the MAXQDA 2022 software (VERBI GmbH) [34].

The research team represented diverse sociocultural backgrounds, aligning with the principles of equity, diversity, and inclusion and contributing to a more nuanced and inclusive interpretation of the data. They included senior and mid- and early-career researchers with several years of experience in qualitative research. Throughout the research process, the researchers engaged in reflexivity and acknowledged how their background, experience, and personal beliefs could shape the interpretation of the data.

## Results

### Characteristics of the Study Participants

The online survey was received by all 144 invited CDOs or responsible officers, of whom 101 (70.1%) returned the questionnaires. Approximately 17.8% (18/101) of the filled-out questionnaires had incomplete responses and were excluded from the analysis, leaving 83 questionnaires for the final analysis.

The mean age of the participants was 50.7 (SD 9.3) years. The majority of the participants were female (48/80, 60%). Approximately 69.9% (n=58) spoke English, 19.3% (n=16) spoke French, 9.6% (n=8) spoke Spanish, and 1.2% (n=1) spoke Russian. In total, 93% (n=77) worked full-time as CDOs or WHO associate members.

Of the 83 participants, 38.6% (n=32) were representatives of the African Region, 8.4% (n=7) were representatives of the Eastern Mediterranean Region, 13.3% (n=11) were representatives of the Region of the Americas, 16.9% (n=14) were representatives of the European Region, 8.4% (n=7) were representatives of the South-East Asia Region, and 14.5% (n=12) were representatives of the Western Pacific Region. The participants were from high-income (n=25, 30.1%), upper-middle–income (n=23, 27.7%), lower-middle–income (n=23, 27.7%), and low-income countries (n=12, 14.5%). More than three-quarters of the respondents (70/83, 84.3%) used a computer to complete the survey. Smartphones were mostly used by respondents from low- or lower-middle–income countries, whereas those from high-income countries (HICs) predominantly used computers.

For the qualitative study, interviews were conducted with 15 participants (n=14, 93.3% female) aged between 34 and 69 years, representing countries of all income levels: 2 (13.3%) from HICs, 3 (20%) from upper-middle–income countries, 6 (40%) from lower-middle–income countries, and 4 (26.7%) from low-income countries. Data saturation was reached after the 10th interview; however, we continued data collection to achieve diversity and at least one representative from all WHO regions.

### Study Findings

#### Overview

**Figure 1:**
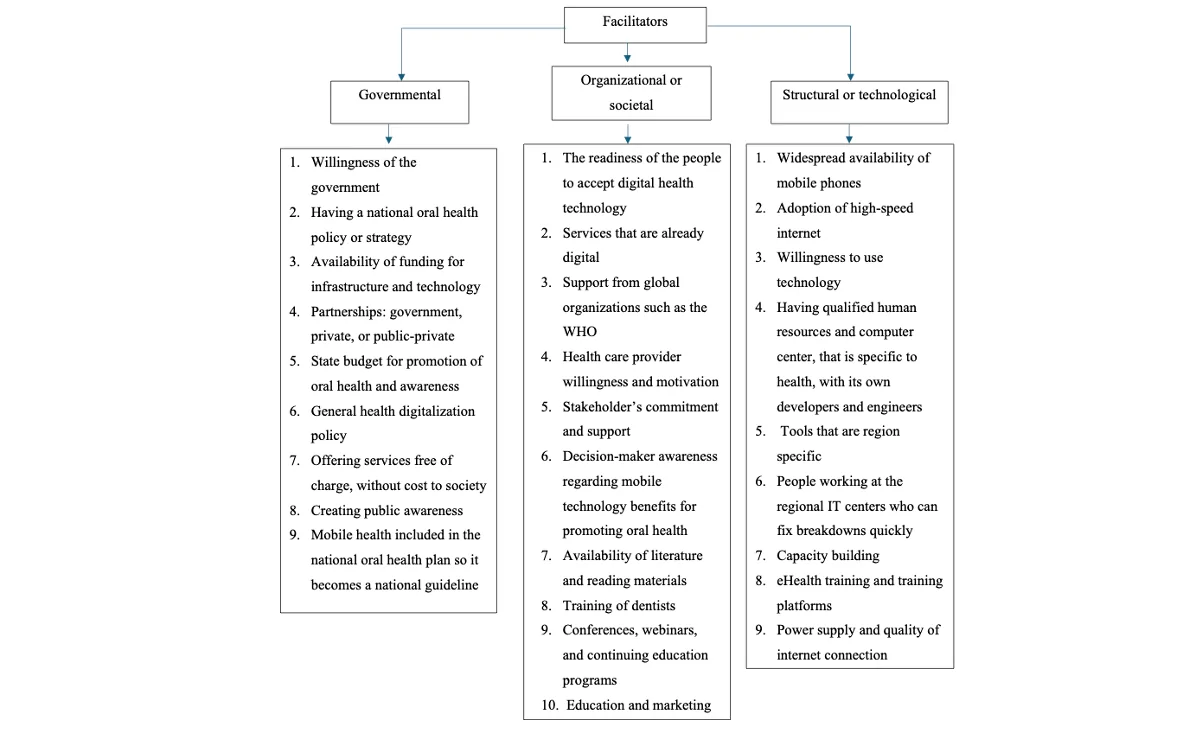
Facilitators of the implementation of digital oral health programs. WHO: World Health Organization.

**Figure 2:**
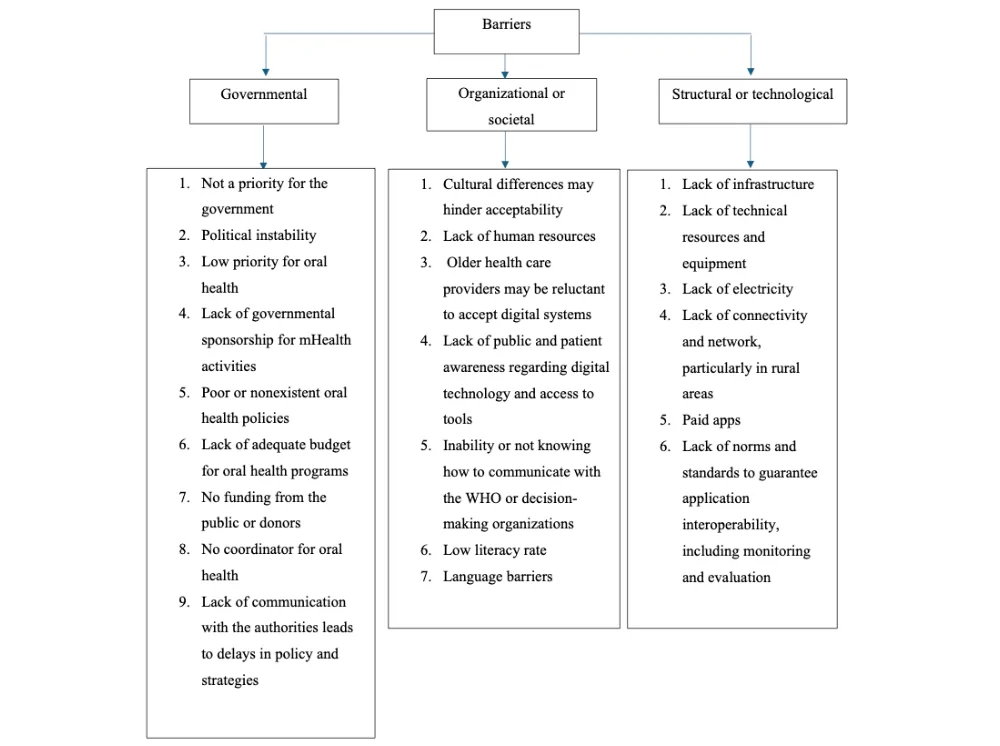
Barriers to the implementation of digital oral health programs. mHealth: mobile health; WHO: World Health Organization.

Both the quantitative and qualitative findings were integrated under 2 broad themes of facilitators and barriers. Perceived facilitators and barriers represented well the macro, meso, and micro domains of e-readiness. The main facilitators and barriers are illustrated in Figure 1 and Figure 2, respectively. Four subthemes (2 per theme) emerged from the analysis.

#### Facilitators

##### Building Digital Health Foundations Through Policy Frameworks and Motivation

Perceived enablers included the presence of national policies and guidelines on eHealth, social acceptance, a desire for digital health, access to necessary digital tools, capacity building on existing e-platforms, and the motivation of health care providers and academic leadership. The theme highlighted the perceived usefulness and positive attitude toward using health technology. Governmental readiness was reflected in the presence of national digital health policies:

We have a very high potential of starting a mOralHealth program because don’t forget that we have something like 143 million active subscribers to mobile phones and there are 173 million people with mobile phones on them. So, it’s almost 90% of the population having access to mobile phones.

CDO 10

According to the survey, 63.9% (n=53) of the participants stated that their country had a national oral health policy, of whom 32.1% (17/53) reported that this policy or strategy clearly referred to digital health to support oral health. Additionally, 60.3% (32/53) of those who indicated that their country had a national oral health policy also had a national digital health or eHealth policy. Digital oral health policies were more common in the WHO European Region. In countries that had an eHealth policy or strategy, 78.1% (25/32) of the participants reported that their countries had adopted a national health information system policy or strategy:

I think the government is very much into digitalization and using artificial intelligence. As you know, digital health is one of the priorities.

CDO 9

With regard to the global implementation of the mOral Health program, the WHO was perceived as a powerful organization that could influence behavioral intentions at the macro level. Moreover, there was a general expectation of support, assistance, and methodological input as 80% (64/80) of the participants reported the need for WHO support to implement e–oral health programs:

The WHO just needs to make very strong recommendations to the government because the government is very, very receptive towards the WHO recommendation. [CDO 8]

The key drivers behind the mOral Health program were working with those who were “the most motivated” and global competition:

It is necessary to work with the most motivated countries, the most motivated providers, the most motivated patient groups. Positive competition must be established.

CDO 2

##### Capacity Building Through Training and Technology

The availability of e-training and e-education, as well as the funding allocated for digital health initiatives, was considered advantageous for the successful implementation and use of digital oral health. The participants expressed their views on the importance of programs and resources to develop skills among health care providers in using digital technologies:

We have a platform for health workers. It’s mostly for their training, like continuing education programs. If we can put it online, if we can integrate it in those programs, that will be one way of implementing it.

CDO 7

In the new generation, there’s almost no problem in adopting mobile health. But for the older generation, we need some training, and we have to socialize the program before it is implemented.

CDO 13

Approximately 46.9% (38/81) of the participants reported that universities or technical colleges provided training for students or professionals on the use of information and communications technologies and eHealth. However, only 34.5% (28/81) reported that institutions (universities or technical colleges) offered training to dentistry students on the use of digital health. More than two-thirds of the respondents (n=60, 72%) reported that they had e-learning courses accredited by continuing medical education or professional licensing bodies.

Technological readiness and health care provider readiness were perceived as strong enablers:

As long as the application is not complicated and you can use very low data, you don’t need high-speed internet. I think we are ready to receive this type of program.

CDO 13

We are able to give information on what we need to do in oral health, in e-health. Whatever the platform that has been set. So, if you have information that is packaged already, then we have a platform.

CDO 2

#### Barriers

##### Limited Prioritization and Resources

Neglected e-oral health priorities and programs; failure to recognize the value of eHealth in health care delivery; and lack of infrastructure, qualified human resources, and funding were highlighted as barriers and societal challenges.

Among the countries included in our study, 45.1% (37/82) had government-sponsored mHealth programs, and only a small percentage, 25.9% (21/81), had a national telehealth policy. In total, 28.9% (24/83) of the participants reported that health authorities provided incentives and guidance for innovation, research, and evaluation regarding eHealth:

We actually do not have an oral health policy in place. So, talking about mOralHealth program would be slightly premature because there is no oral health policy or strategy that we are following.

CDO 10

The scarcity of financial support was one of the main concerns regarding digital oral health implementation. The survey revealed that 31.7% (26/82) of the participants reported having no financial support (public, private, both, or from donors) available for the implementation of their national digital oral health programs. Among all participants, 36.6% (30/82) reported having received funding from the public sector, 19.5% (16/82) from the private sector, 18.3% (15/82) from donors, and 18.3% (15/82) from public-private partnerships. As respondents could report more than one source of funding, these categories were not mutually exclusive. Furthermore, 10.9% (9/82) of the participants reported receiving all 4 types of funding. Furthermore, 39% (32/82) of the participants reported that their national digital health policy funding did not support oral health.

Among the participants who reported receiving public sector funding (n=30), 9 (30%) were from the European Region, representing the highest percentage among other regions. Among the participants who reported receiving financial support through private organizations (n=16) or public-private partnerships (n=15), 11 (68.7%) and 8 (53.3%) were from high-income countries, respectively.

As far as the funding is concerned, again, let me remind you that we spend less than 3% or 2% of our capital, our GDP, on health. And since oral health is very low on priority, there is practically nothing left for oral health when it comes to a national policy. So funding, again, is an issue as far as the country’s readiness.

CDO 10

Inadequate technological access, infrastructure, and integration with existing services; the limited support for implementing mHealth initiatives; and the lack of norms and standards to guarantee application interoperability were perceived as important barriers. At the meso level, issues with structural and technological readiness, technical interoperability between existing systems, and a lack of equipment or connectivity were also identified as barriers:

There are definitely issues related to specifically Wi-Fi and internet connectivity where everyone seems to have some kind of cellphone or tablet kind of mechanism for social media access. When it comes to actual connectivity data, etc., it costs money for them to download information, download material.

CDO 11

Sometimes in big cities, we can implement this type of program without any problem. But when we go to the rural area, to the remote island, they cannot do this because there is no signal or enough internet to do that.

CDO 13

A lot of healthcare workers are keen and willing to be part of a program. But I think again. If there are problems, say, with the ongoing lack of connectivity, when they need to get in touch with a consultant and if they cannot at that moment or if they do not have efficient and ready ICT support...the healthcare providers lose interest if there isn’t a good two-way feedback when we implement these programs.

CDO 11

##### Hesitation and Uncertainty

The lack of demand for eHealth programs among health professionals was a significant barrier for upper-middle–, low-middle–, and low-income countries. Moreover, only approximately one-quarter of all participants (n=18, 21.6%) reported that health authorities provided regulatory oversight for mHealth apps in terms of quality, safety, and reliability. Additionally, only one-third (n=24, 29%) of all participants stated that health authorities offered incentives and guidance for innovation, research, and evaluation regarding eHealth.

Furthermore, concerns related to the lack of evidence on the effectiveness and cost-effectiveness of programs, as well as a lack of confidence in digital and data management (eg, cyber risks), were expressed as barriers by 73.8% (59/80), 73% (57/78), and 69.6% (55/79) of the participants, respectively:

My comment would be around the availability of reading materials that could help to get people prepared. Is there literature people can access online to familiarize themselves with what this is and what it involves, so that we can share them to disseminate and start getting that feeling with people.

CDO 7

## Discussion

### Principal Findings

This study identified key factors influencing the adoption of digital oral health in countries belonging to the 6 WHO regions from the decision-makers’ perspective. The key enablers identified were the presence of national eHealth policies, availability of funding for infrastructure and technology, and health care provider willingness. On the other hand, the barriers identified were the lack of governmental support for digital health, lack of partnerships, neglected oral health programs, lack of infrastructure and funding, and inadequate human resources. This study revealed that the frequency of these barriers was higher in low- and middle-income countries (LMICs), where policy gaps, competing health care priorities, and limited resources often exacerbate these issues compared to HICs.

### Comparison With Prior Work

Our findings are in agreement with those of previous systematic reviews [35,36] that have pointed to insufficient infrastructure, outdated equipment, technology gaps, and human resource challenges as major barriers to mHealth implementation in LMICs. Additionally, health care executives in Ethiopia have emphasized further challenges, including economic inflation, low digital literacy, and a lack of system interoperability as significant challenges to the adoption of mHealth solutions [37]. These barriers collectively pose significant threats to the performance of the health workforce, undermining their efforts to deliver high-quality care and further perpetuating health disparities.

Underuse and slow progression of digital health initiatives and mHealth programs despite the growing adoption of mobile phones and recognition of their potential benefits was a finding in LMICs. One key factor contributing to this issue could be the low mobile internet penetration in these regions, which stands at 69% and only 48% when China is excluded despite an estimated 2.9 billion current users of mobile phones [38,39]. This is in sharp contrast to 87% mobile internet penetration in HICs [39]. The lack of mobile internet access can prevent the effective use of mHealth tools that depend on real-time communication, data transmission, and access to digital health platforms. Consequently, the government’s role is crucial to improve the telecommunications infrastructure and enhance mobile internet access by making it more affordable through low costs of mobile devices and data services [40].

Digital technology offers an opportunity to enhance education, incentives, aid, supervision, and support for universal health coverage [41]. However, these efforts require adequate funding, the lack of which was reported as a significant barrier in our study and has also been reported as a barrier in other research [35,36], affecting both HICs and LMICs. The successful implementation of digital health interventions requires investment not only in technology but also in operational costs; training for dental health care providers; and the development of health care workflows, including data management and security systems. Securing sustained support from the government and relevant local funders and institutions is crucial for the success of digital interventions [42]. Moreover, to facilitate both scaling up and financial sustainability, diverse funding sources, such as private sector investments and public-private partnerships, should be sought rather than relying on a single source [40,43].

In addition to funding, proactive government engagement and the development of clear policies are essential, with well-defined roles and responsibilities at all levels, from government bodies to health care managers and dental health care providers [35]. Engaging local stakeholders and collaborating with mobile technology developers early in the design phase can help ensure that the interventions are both suitable and embraced [35]. Furthermore, strong partnerships between the national and local governments, nongovernmental organizations, private entities, and global organizations such as the WHO must be established for active leadership toward the successful implementation and long-term sustainability of the programs [40]. Within routine dental practices, ensuring successful implementation of digital health would require reforms in dental education toward the development of relevant competencies, positive attitudes, and behavioral intentions, ensuring easy access to digital technologies and establishing supportive policies and regulatory frameworks [44,45].

### Strengths and Limitations

To our knowledge, this is the first study conducted in partnership with the WHO on global e–oral health readiness that involved participants from many countries working in different settings and with various profiles. The results of this study provide a basis for comparison and future planning. The mixed methods design used in this study enabled the combination of 2 complementary datasets to generate meaningful information and gain a strong understanding of factors that can be considered in developing a global strategic plan and improving the success of mOral Health implementation. However, this study has some limitations. First, while the participation rate was acceptable, the survey was self-administered, which may have resulted in an over- or underestimation of information. Second, many surveys were incomplete, resulting in the impossibility of analyzing nonrespondents. However, the purposeful sampling technique for the qualitative study provided rich data due to the inclusion of diverse participants from the 6 WHO regions. Third, the participants in this study represented WHO member countries from the 6 WHO regions. Therefore, the results may not be generalizable to nonmember countries, whose national health and digital health policies might differ from those of the countries included in our study.

### Conclusions

This study identified key barriers to and enablers of implementing digital oral health programs in WHO member states through an e-readiness assessment across multiple levels. The analysis revealed gaps in funding support, the establishment of norms and regulations, and the availability of robust infrastructure. As readiness is essential for the successful adoption of new policies, practices, and technologies, its systematic assessment is crucial for integrating digital oral health into oral health care systems and practice to advance global oral health outcomes. Governments play a central role in this process by developing concrete policies, investing in infrastructure, and allocating adequate budgets for e–oral health technologies to overcome the limitations of traditional health care delivery models.

## Appendix

Multimedia Appendix 1 GRAMMS checklist.

Multimedia Appendix 2 Questionnaire related to facilitators and barriers.

Multimedia Appendix 3 Interview guide.

## Abbreviations

AI: artificial intelligence
CDO: chief dental officer
GRAMMS: Good Reporting of a Mixed Methods Study
HIC: high-income country
LMIC: low- and middle-income country
mHealth: mobile health
WHO: World Health Organization

## Data Availability

The datasets generated or analyzed during this study are available from the corresponding author on reasonable request.

## References

[1] Meskó B, Drobni Z, Bényei É, Gergely B, Győrffy Z (2017). Digital health is a cultural transformation of traditional healthcare. Mhealth.

[2] Nundy S, Cooper LA, Mate KS (2022). The quintuple aim for health care improvement: a new imperative to advance health equity. JAMA.

[3] Coleman K, Wagner E, Schaefer J, Reid R, LeRoy L (2016). Redefining primary care for the 21st century. Agency for Healthcare Research and Quality.

[4] Peres MA, Macpherson LM, Weyant RJ, Daly B, Venturelli R, Mathur MR, Listl S, Celeste RK, Guarnizo-Herreño CC, Kearns C, Benzian H, Allison P, Watt RG (2019). Oral diseases: a global public health challenge. Lancet.

[5] Wang J, Wang B, Liu YY, Luo YL, Wu YY, Xiang L, Yang XM, Qu YL, Tian TR, Man Y (2024). Recent advances in digital technology in implant dentistry. J Dent Res.

[6] Lee SJ, Poon J, Jindarojanakul A, Huang CC, Viera O, Cheong CW, Lee JD (2025). Artificial intelligence in dentistry: exploring emerging applications and future prospects. J Dent.

[7] Mahdavi A, Atlasi R, Naemi R (2022). Teledentistry during COVID-19 pandemic: scientometric and content analysis approach. BMC Health Serv Res.

[8] Gurgel-Juarez N, Torres-Pereira C, Haddad AE, Sheehy L, Finestone H, Mallet K, Wiseman M, Hour K, Flowers HL (2022). Accuracy and effectiveness of teledentistry: a systematic review of systematic reviews. Evid Based Dent.

[9] Kengne Talla P, Allison P, Bussières A, Rodrigues A, Bergeron F, Giraudeau N, Emami E (2025). Teledentistry for improving access to, and quality of oral health care: overview of systematic reviews and meta-analyses. J Med Internet Res.

[10] Cresswell K, Sheikh A (2013). Organizational issues in the implementation and adoption of health information technology innovations: an interpretative review. Int J Med Inform.

[11] Desveaux L, Soobiah C, Bhatia RS, Shaw J (2019). Identifying and overcoming policy-level barriers to the implementation of digital health innovation: qualitative study. J Med Internet Res.

[12] (2023). Draft global oral health action plan (2023–2030). World Health Organization.

[13] (2021). Global strategy on digital health 2020-2025. World Health Organization.

[14] Jagde AK, Shrivastava R, Feine J, Emami E (2021). Patients' e-readiness to use e-health technologies for oral health. PLoS One.

[15] Nilsen ER, Stendal K, Gullslett MK (2020). Implementation of eHealth technology in community health care: the complexity of stakeholder involvement. BMC Health Serv Res.

[16] Tossaint-Schoenmakers R, Versluis A, Chavannes N, Talboom-Kamp E, Kasteleyn M (2021). The challenge of integrating eHealth into health care: systematic literature review of the donabedian model of structure, process, and outcome. J Med Internet Res.

[17] Khoja S, Scott RE, Casebeer AL, Mohsin M, Ishaq AF, Gilani S (2007). e-Health readiness assessment tools for healthcare institutions in developing countries. Telemed J E Health.

[18] Mauco KL, Scott RE, Mars M (2020). Validation of an e-health readiness assessment framework for developing countries. BMC Health Serv Res.

[19] Yusif S, Hafeez-Baig A, Soar J (2020). An exploratory study of the readiness of public healthcare facilities in developing countries to adopt health information technology (HIT)/e-Health: the case of Ghana. J Healthc Inform Res.

[20] Jivraj A, Barrow J, Listl S (2022). Value-based oral health care: implementation lessons from four case studies. J Evid Based Dent Pract.

[21] O'Cathain A, Murphy E, Nicholl J (2008). The quality of mixed methods studies in health services research. J Health Serv Res Policy.

[22] Ivankova NV, Creswell JW, Stick SL (2006). Using mixed-methods sequential explanatory design: from theory to practice. Field Methods.

[23] Palinkas LA, Aarons GA, Horwitz S, Chamberlain P, Hurlburt M, Landsverk J (2011). Mixed method designs in implementation research. Adm Policy Ment Health.

[24] Creswell JW (2003). Research Design: Qualitative, Quantitative, and Mixed Methods Approaches.

[25] Venkatesh V, Davis FD (2000). A theoretical extension of the technology acceptance model: four longitudinal field studies. Manag Sci.

[26] Indicator metadata registry list. World Health Organization.

[27] (2016). Atlas of eHealth country profiles: the use of eHealth in support of universal health coverage: based on the findings of the third global survey on eHealth 2015. World Health Organization.

[28] Palinkas LA, Horwitz SM, Green CA, Wisdom JP, Duan N, Hoagwood K (2015). Purposeful sampling for qualitative data collection and analysis in mixed method implementation research. Adm Policy Ment Health.

[29] Dillman DA (2006). Why choice of survey mode makes a difference. Public Health Rep.

[30] Saunders B, Sim J, Kingstone T, Baker S, Waterfield J, Bartlam B, Burroughs H, Jinks C (2018). Saturation in qualitative research: exploring its conceptualization and operationalization. Qual Quant.

[31] Ross RK, Breskin A, Westreich D (2020). When is a complete-case approach to missing data valid? The importance of effect-measure modification. Am J Epidemiol.

[32] Creswell JW, Creswell JD (2017). Research Design: Qualitative, Quantitative, and Mixed Methods Approaches.

[33] Korstjens I, Moser A (2018). Series: practical guidance to qualitative research. Part 4: trustworthiness and publishing. Eur J Gen Pract.

[34] VERBI software. MAXQDA.

[35] Aranda-Jan CB, Mohutsiwa-Dibe N, Loukanova S (2014). Systematic review on what works, what does not work and why of implementation of mobile health (mHealth) projects in Africa. BMC Public Health.

[36] Kruse C, Betancourt J, Ortiz S, Valdes Luna SM, Bamrah IK, Segovia N (2019). Barriers to the use of mobile health in improving health outcomes in developing countries: systematic review. J Med Internet Res.

[37] Aboye GT, Simegn GL, Aerts J (2024). Assessment of the barriers and enablers of the use of mHealth systems in sub-Saharan Africa according to the perceptions of patients, physicians, and health care executives in Ethiopia: qualitative study. J Med Internet Res.

[38] Bahia K (2020). The state of mobile internet connectivity report 2020. GSMA Intelligence.

[39] Bahia K (2023). The state of mobile internet connectivity 2023. GSMA Intelligence.

[40] Barkman C, Weinehall L (2017). Policymakers and mHealth: roles and expectations, with observations from Ethiopia, Ghana and Sweden. Glob Health Action.

[41] Long LA, Pariyo G, Kallander K (2018). Digital technologies for health workforce development in low- and middle-income countries: a scoping review. Glob Health Sci Pract.

[42] McCool J, Dobson R, Muinga N, Paton C, Pagliari C, Agawal S, Labrique A, Tanielu H, Whittaker R (2020). Factors influencing the sustainability of digital health interventions in low-resource settings: lessons from five countries. J Glob Health.

[43] (2015). The MAPS toolkit. World Health Organization.

[44] Schnitzler C, Bohnet-Joschko S (2025). Technology readiness drives digital adoption in dentistry: insights from a cross-sectional study. Healthcare (Basel).

[45] Galazzi A, Fonda F, Chiappinotto S, Justi L, Sønderskov Frydensberg M, Lehmann Boesen R, Macur M, de San Pedro M, Reixach Espaulella E, Palese A (2025). Recommendations to promote the digital healthcare transformation in the clinical practice: findings from an international consensus development method. BMC Health Serv Res.

