# Correction: Facilitators of and Barriers to Global Digital Oral Health: Mixed Methods Study

**DOI:** 10.2196/112225

**Published:** 2026-09-29

**Authors:** Elham Emami, Pascaline Kengne Talla, Camille Inquimbert, Nicolas Giraudeau

**Affiliations:** 1 Faculty of Dental Medicine and Oral Health Sciences McGill University Montreal, QC Canada; 2 Centre d’études politiques et sociales, Centre national de la recherche scientifique University of Montpellier Montpellier France

In “Facilitators of and Barriers to Global Digital Oral Health: Mixed Methods Study” [1], the authors made the following changes to the Results section.

In the Characteristics of the Study Participants subsection, the following sentence was revised:

More than three-quarters of the respondents (80/83, 84.3%) used a computer to complete the survey.

The sentence now reads:

More than three-quarters of the respondents (70/83, 84.3%) used a computer to complete the survey.

In the Barriers subsection under Study Findings, the following sentences were revised:

Among those who did have funding, 36.6% (30/82) received it from the public sector, 19.5% (16/82) received it from the private sector, 18.3% (15/82) received it from donors, and 18.3% (15/82) received it from public-private partnerships.Only 10.9% (9/82) of the participants reported all 4 types of funding.

The sentences now read:

Among all participants, 36.6% (30/82) reported having received funding from the public sector, 19.5% (16/82) from the private sector, 18.3% (15/82) from donors, and 18.3% (15/82) from public-private partnerships. As respondents could report more than one source of funding, these categories were not mutually exclusive. Furthermore, 10.9% (9/82) of the participants reported receiving all 4 types of funding.

In the same subsection, the following sentences were revised:

Public funding was the most commonly reported funding type among participants from countries in the European Region (9/30, 30%). Approximately 68.7% (11/16) of the higher-income countries had adequate financial resources through private organizations or public-private partnerships (8/15, 53.3%):

The sentences now read:

Among the participants who reported receiving public sector funding (n=30), 9 (30%) were from the European Region, representing the highest percentage among other regions. Among the participants who reported receiving financial support through private organizations (n=16) or public-private partnerships (n=15), 11 (68.7%) and 8 (53.3%) were from high-income countries, respectively.

The correction will appear in the online version of the paper on the JMIR Publications website together with the publication of this correction notice. Because this was made after submission to PubMed, PubMed Central, and other full-text repositories, the corrected article has also been resubmitted to those repositories.
