# Supplementary material for: Facilitators of and Barriers to Global Digital Oral Health: Mixed Methods Study
Source: J Med Internet Res. 2026 Apr 30;28:e76236. doi: 10.2196/76236 (PMC13176808; doi:10.2196/76236)
Supplement: Multimedia Appendix 1 [file jmir_v28i1e76236_app1.docx]

**Good Reporting of a Mixed Methods Study (GRAMMS) checklist**

| **Guideline** | **Page** |
| --- | --- |
| Describe the justification for using a mixed methods approach to the research question | Pages 6 |
| Describe the design in terms of the purpose, priority and sequence of methods | Pages 6 |
| Describe each method in terms of sampling, data collection and analysis | Pages 6-9 |
| Describe where integration has occurred, how it has occurred and who has participated in it | Pages 10 |
| Describe any limitation of one method associated with the present of the other method | Pages 17,19 |
| Describe any insights gained from mixing or integrating methods | Page 17,19 |
