# Supplementary material for: Facilitators of and Barriers to Global Digital Oral Health: Mixed Methods Study
Source: J Med Internet Res. 2026 Apr 30;28:e76236. doi: 10.2196/76236 (PMC13176808; doi:10.2196/76236)
Supplement: Multimedia Appendix 2 [file jmir_v28i1e76236_app2.pdf]

THE USE OF DIGITAL HEALTH IN SUPPORT OF ORAL HEALTH

DIGITAL HEALTH FOUNDATIONS

National policies or strategies

Does your country have a national oral health policy or strategy?

Yes
No
Don’t know

If yes, does your national oral health policy or strategy clearly refer to the use of Digital Health to support oral health?

Yes
No
Don’t know

If yes, does your country have a national Digital Health policy or strategy (1)?

Yes
No
Don’t know

(1): A national eHealth policy or strategy lays out the vision and objectives to promote the use of information and communication technologies (ICT) specifically for the health sector. For the purpose of this survey, policy and strategy are used interchangeably, although this is not strictly correct.

If yes, does your country have a national health information system (HIS) policy or strategy (2)?

Yes
No; No – no separate policy or strategy but is included in the national eHealth policy or strategy;
Don’t know

(2): A national health information system policy or strategy lays out the vision and objectives for a national system to meet the health information needs of the country. It can include such elements as vital registration, notifiable diseases, private sector information such as insurance, and patient confidentiality guidelines.

Funding

Public funding is financial support provided by government and can come from national, regional or district-levels.
Is this type of funding available for Digital Oral Health programs?

Yes
No
Don’t know

Private or commercial funding is financial or in-kind support provided by the private or commercial sector.
Is this type of funding available for Digital Oral Health programs?

Yes
No
Don’t know

Donor/non-public development funding is financial or in-kind support provided by development agencies, development banks, foundations or other non-public funding bodies for development work. It can be international, regional or national.
Is this type of funding available for Digital Oral Health programs?

Yes
No
Don’t know

Public-private partnerships are joint ventures between public organizations and private sector companies to work together to achieve a common goal.
Is this type of funding available for Digital Oral Health programs?

Yes
No
Don’t know

Policy or strategy implementation funding is financial support specifically provided to help with the implementation of a national Digital Health policy or strategy.
Is there special funding allocated for the implementation of your country’s Digital Health policy or strategy?

Yes

No; No – a national Digital Health policy or strategy does not exist
Don’t know

Capacity building - Human resources knowledge and skills

Pre-service training - Health sciences students

Do any tertiary institutions (universities or technical colleges) in your country provide students of dentistry training on the use of Digital Health?

Yes
No
Don’t know

Do tertiary institutions (universities or technical colleges) in your country teach students of health sciences in the use of digital health?

Yes
No
Don’t know

In-service training - Health professionals

Do any institutions or associations in your country offer in-service training in the use of digital health as part of the continuing education of dental professionals?

Yes
No
Don’t know

mHEALTH

Are any government-sponsored mHealth programs being conducted in your country?

Yes
No
Don’t know

What is the role or function of health authorities in your country with respect to the development and adoption of mHealth? Check as many as apply.
Regulating mobile devices and software for quality, safety and reliability
Promoting standards and interoperability
Providing guidance for privacy and security
Providing oversight and enforcement of data ownership

Promoting the development and adoption of mHealth in the health sector

No role
Don't know
Other

Other, please specify:

Which policies or strategies guide your mHealth programs?
National Digital Health policy or strategy
National mHealth policy or strategy
National telehealth policy or strategy
No specific guidance available
Don't know
Other

Other, please specify:

Mobile applications

Is there an entity in your country responsible for the regulatory oversight of mobile health apps for quality, safety and reliability?

Yes
No
Don't know

Is there an entity in your country that provides incentives and guidance for innovation, research and evaluation of health apps?

Yes
No
Don't know

TELEHEALTH

National telehealth policy or strategy

Does your country have a dedicated national telehealth policy or strategy?
Yes
No – but telehealth is referred to in the overall national Digital Health policy or strategy;
No

Don’t know

If Yes; No – but telehealth is referred to in the overall national Digital Health policy or strategy", does your policy or strategy include objectives that address how telehealth can contribute to oral health?

Yes
No
Don’t know

Target group: Health professionals (in-service)

Is eLearning used for the in-service training of health professionals in your country?

Yes
No
Don’t know

If yes, are eLearning courses accredited by continuing medical education (CME) or professional licensing bodies in your country?

Yes
No
Don’t know

BARRIERS TO IMPLEMENTING DIGITAL HEALTH PROGRAMS

Barriers to implementing eHealth programs

Capacity - lack of trained human resources
Infrastructure - lack of equipment and/or connectivity.
Interoperability - lack of norms and standards to guarantee applications interoperability
Funding - lack of funding to develop and support Digital Health programs.
Effectiveness - lack of evidence on effectiveness of programs.
Cost-effectiveness - lack of evidence on cost-effectiveness of programs.
Demand - lack of demand for Digital Health programs by health professionals or target groups.
Confidence - lack of confidence in digital and data management (cyber risks, etc.)
Legal - lack of legislation or regulations covering Digital Health programs.
Policy - national policies do not recognize Digital Health in health services delivery.
Priorities - competing health system priorities.

Not a barrier
Moderate barrier
Strong barrier

DIGITAL HEALTH NETWORKS

Digital Health support and advocacy
Please provide details on professional associations or industry-related groups which provide leadership in your country in Digital Health:

Digital Health national governance and guidance
Please provide details on any entities in your country, such as Digital Health task force, advisory board, or similar, that provide advice and guidance in areas such as policy/strategy, program implementation, legal issues, and evaluation in Digital Health? (Up to three entities):

Do you need support from the WHO headquarters to implement mOral Health actions?
Yes; No

If yes, please confirm your country
