# Supplementary material for: Facilitators of and Barriers to Global Digital Oral Health: Mixed Methods Study
Source: J Med Internet Res. 2026 Apr 30;28:e76236. doi: 10.2196/76236 (PMC13176808; doi:10.2196/76236)
Supplement: Multimedia Appendix 3 [file jmir_v28i1e76236_app3.docx]

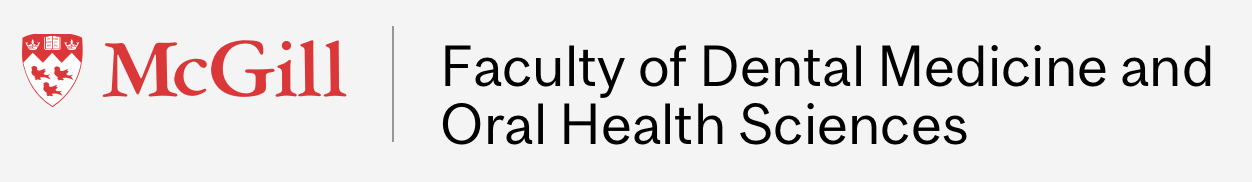


Faculty of Dental Medicine and Oral Health Sciences

McGill University,

2001 Ave McGill College,

Montreal, QC H3A 1G1

**INTERVIEW GRID**

**Date:**

**Time and Place:**

**Numero of interview:**

1. **INTERVIEW OPENING**

- Greet and welcome the interviewee; introduce yourself (interviewer / position) and make the applicant feel comfortable.
- Explain the purpose of the interview and inform the participant about the duration of the interview; allow interviewee to read consent.
- Invite the interviewee to clarify any doubts regarding the interview and obtain online signature on copies of consent form.
- Describe the interview process.
- Before starting, talk to participant regarding confidentiality.

Mention that:

- - - Your anonymity will be respected.
    - Your name will not be revealed or published in any document.
    - You can withdraw any time (if you feel uncomfortable).
    - Install the tape recorder (make test of recording).

**B) CONDUCTING THE INTERVIEW**

- Begin with unstructured, open questions to encourage the spontaneity.
- Give brief description at the end of each section to make sure that we understood well and to give the opportunity to the interviewee to complete or to modify their statements (reformulation).

**BACKGROUND**

Some questions for the qualitative component:

1. How would you describe the state of governmental readiness, or the presence of relevant policies and funding for implementation of the mOralHealth programme?
2. What do you think about organizational readiness (presence of policies & management support) for implementation of the mOralHealth programme?
3. The interaction of health care institutions with their government and communities can have an impact on implementation of the mOralHealth programme. From your point of view, what is the state of societal readiness implementation of the mOralHealth programme?
4. What is the impact of technological / infrastructural readiness (skilled human resources, ICT support, quality ICT infrastructure, and power supply) on implementation of the mOralHealth programme?
5. To what extent do you think healthcare provider readiness (healthcare providers’ personal experience, primarily their perception and receptiveness towards the use of e-health technology) can partially or totally influence implementation of the mOralHealth programme?
6. Do you think the engagement readiness (perceived benefits as well as negative impacts of e-health, acceptance of training for e-health among members of a community) can have an impact on implementation of the mOralHealth programme?
7. What are the factors of core readiness (the extent to which members of a community are dissatisfied with the current status of their healthcare service provision, see e-health as a solution, and express their need and preparedness for e-health services) which can influence implementation of the mOralHealth programme?
8. What do you think about the public / patient readiness (public and patients’ awareness, affordability, access to e-health services, personal experiences on their perception and receptiveness towards the use of e-health technology) on implementation of the mOralHealth programme?
9. Regarding these types of readiness, how would you prioritize them? What are the top 5 readiness components influencing implementation of the mOralHealth programme?
10. Do you know any strategies to improve implementation of the mOralHealth programme?
11. **Closing THE INTERVIEW**
12. Is there anything else that seems important to you and that you would like to talk about? Any comment?
13. Thank participant for their time and contribution.
14. Ask if the participant can be contacted later if necessary.
15. **AFTER THE INTERVIEW**
16. Verify if the tape recording has worked.
17. Write down any observations made during the interview.
